# Over-diagnosis of malaria by microscopy in the Kilombero Valley, Southern Tanzania: an evaluation of the utility and cost-effectiveness of rapid diagnostic tests

**DOI:** 10.1186/1475-2875-12-159

**Published:** 2013-05-10

**Authors:** Kelly Harchut, Claire Standley, Andrew Dobson, Belia Klaassen, Clotilde Rambaud-Althaus, Fabrice Althaus, Katarzyna Nowak

**Affiliations:** 1 Princeton University, 106A Guyot Hall, Princeton, NJ 08544, USA; 2 IST Clinic, PS Box 2651, Dar es Salaam, Tanzania; 3 Swiss Tropical and Public Health Institute, Socinstrasse 57, Basel, CH 4051, Switzerland

## Abstract

**Background:**

Early and accurate diagnosis of febrile patients is essential to treat uncomplicated malaria cases properly, prevent severe malaria, and avert unnecessary anti-malarial treatments. Improper use of anti-malarials increases the risk of adverse drug reaction and the evolution of drug/parasite resistance. While microscopy is the most common form of malaria diagnosis, concerns over its accuracy have prompted the incorporation of malaria rapid diagnostic tests (RDTs) into many national malaria control programmes.

**Methods:**

Over a three-month period, a direct comparison between microscopy and RDTs was made in a rural, private dispensary in the Kilombero Valley, Morogoro District, southern Tanzania, with the aim of estimating the extent of malaria over-diagnosis and over-treatment with anti-malarials. The study cohort was made up of patients referred by the dispensary’s clinician for malaria testing. One hundred percent of patients approached agreed to participate in this study and were then tested using both microscopy and RDTs. Using the results from the comparison of the two tests at this dispensary, the potential cost effectiveness of introducing RDTs to a neighbouring public health centre was estimated on the basis of this centre’s past malaria records spanning December 2007 to August 2011.

**Results:**

At the private dispensary, the apparent prevalence of malaria was 78% based on microscopy whereas the true prevalence, calculated using RDTs as the gold standard, was estimated at 14%. This discrepancy indicates that when using microscopy as the sole diagnostic test, malaria is being over-diagnosed by approximately a factor of five in this setting. At the public clinic, apparent malaria prevalence based on microscopy was 74%. If similar rates of over-diagnosis are assumed, 5,285 patients of the 6,769 patients positively diagnosed with malaria using microscopy were likely given unnecessary anti-malarials, and their true cause of illness was not addressed. The introduction of RDTs to the public clinic would be highly cost-efficient, with an estimated net saving of over 96 USD/month.

**Conclusions:**

Compared with RDTs, microscopy led to almost four out of five patients being over-diagnosed with malaria in this rural part of Tanzania. A policy that encompasses both the private and public sectors of health care is needed to ensure quality diagnostic testing for febrile patients. With estimated prevalence at 14%, RDT introduction is recommended given WHO findings that RDTs are predicted to be cost-effective in prevalence areas of less than 20%. The use of RDTs in malaria diagnosis would not only reduce government spending but would prove beneficial to ensuring appropriate care and treatment of febrile illness.

## Background

Malaria is currently endemic in 106 countries where it leads to an estimated 216 million cases per year and 655,000 deaths, the majority of which are in sub-Saharan Africa [1]. Recent large-scale efforts are underway in many malaria-endemic regions to reduce the morbidity and mortality associated with malaria; improving access to effective treatment is a significant part of these initiatives [2]. As a result of these efforts, malaria cases and malaria-related deaths in mainland Tanzania have been reduced by up to 50% in the past decade [3].

The extensive deployment of anti-malarial drugs has provided strong selective pressure on human malaria parasites to evolve mechanisms of resistance [4]. This is substantiated in the rapid spread of *Plasmodium falciparum* resistance to chloroquine [5], and the efforts made to limit drug-resistant malaria through the use of artemisinin-based combination therapy (ACT) over artemisinin monotherapy [6,7]. However, other factors also contribute to resistance, and accurate diagnosis of malaria can help ensure that ACT is not over-prescribed. Based on the presumption that most fever cases in endemic areas were the result of malaria, WHO once recommended presumptive treatment for malaria, particularly for children under the age of five [1]. However, when unnecessary treatments are dosed or taken improperly, particularly in regions of high malaria endemicity where many people suffer from frequent subclinical malaria infections, resistance can rapidly emerge. Moreover, indiscriminate treatment of most febrile illness, such as malaria, results in the true cause of illness failing to be identified if the patient is in fact non-malarial. Documenting the extent to which ACT is used unnecessarily is thus crucial to curbing drug resistance and improving patient care.

In health facilities with laboratories, simple light microscopy examinations of Giemsa-stained blood films are the most widely practiced method for malaria diagnosis [8]. Advantages of microscopy include differentiation between species, quantification of parasite density, and ability to distinguish clinically important asexual parasite stages from gametocytes – the sexual stage of the parasite, which may persist without causing symptoms [9]. Despite these advantages, in many parts of sub-Saharan Africa, routine microscopy is known to be of low quality due to poor training, low skill level of laboratory staff, poor infrastructure, inadequacy of equipment and reagents, and unreliable electricity [8,10]. These challenges may result in laboratory technicians’ over-diagnosis of malaria, which may be the result from previous WHO recommendations based on the assumption that it is safer to treat non-malarial patients with anti-malarials than missing a true case of malaria [11]. Therefore, even though microscopy can yield accurate and specific malaria diagnosis, in environments with limited resources and funding, such as in sub-Saharan Africa, its use can be associated with routine over-diagnosis of malaria [12].

Malaria rapid diagnostic tests (RDTs), which require minimal training, have begun to be used for routine malaria testing in a number of African countries and have proved to have high sensitivity and specificity [8,13]. Requiring only a drop of blood, RDTs detect antigens specific to *Plasmodium* species. Some RDTs can differentiate between malaria species while others test only for antigens specific to *P*. *falciparum*.

According to WHO, approximately 40% of malaria patients worldwide seek treatment for malaria in the private sector, which includes regulated health facilities, pharmacies and other drug outlets [14]. As such, the Affordable Medicines Facility for malaria (AMFm), a “factory-gated” subsidy for ACT launched in 2010, aims to increase the availability of effective, artemisinin-combination anti-malarials in both the public and private sectors [15]. AMFm increases the availability of ACTs, in part because a subsidy is provided. However, this program has very little focus on accurate diagnosis of patients with malaria. It is therefore important that both the public and private sectors are considered in research investigating the implications of diagnostic tools on public health outcomes.

Within the setting of the Kilombero Valley of southern Tanzania, a malaria hotspot, the aim of this study was to conduct a direct comparison between microscopy and RDTs at a private clinic in order to estimate the percentage of the population seeking anti-malarial treatment in the private sector that is actually infected with malaria. Then, using results from this comparison, this study aimed to estimate the cost-effectiveness of RDTs for malaria diagnosis in this rural area.

## Methods

### Study site

**Figure 1:**
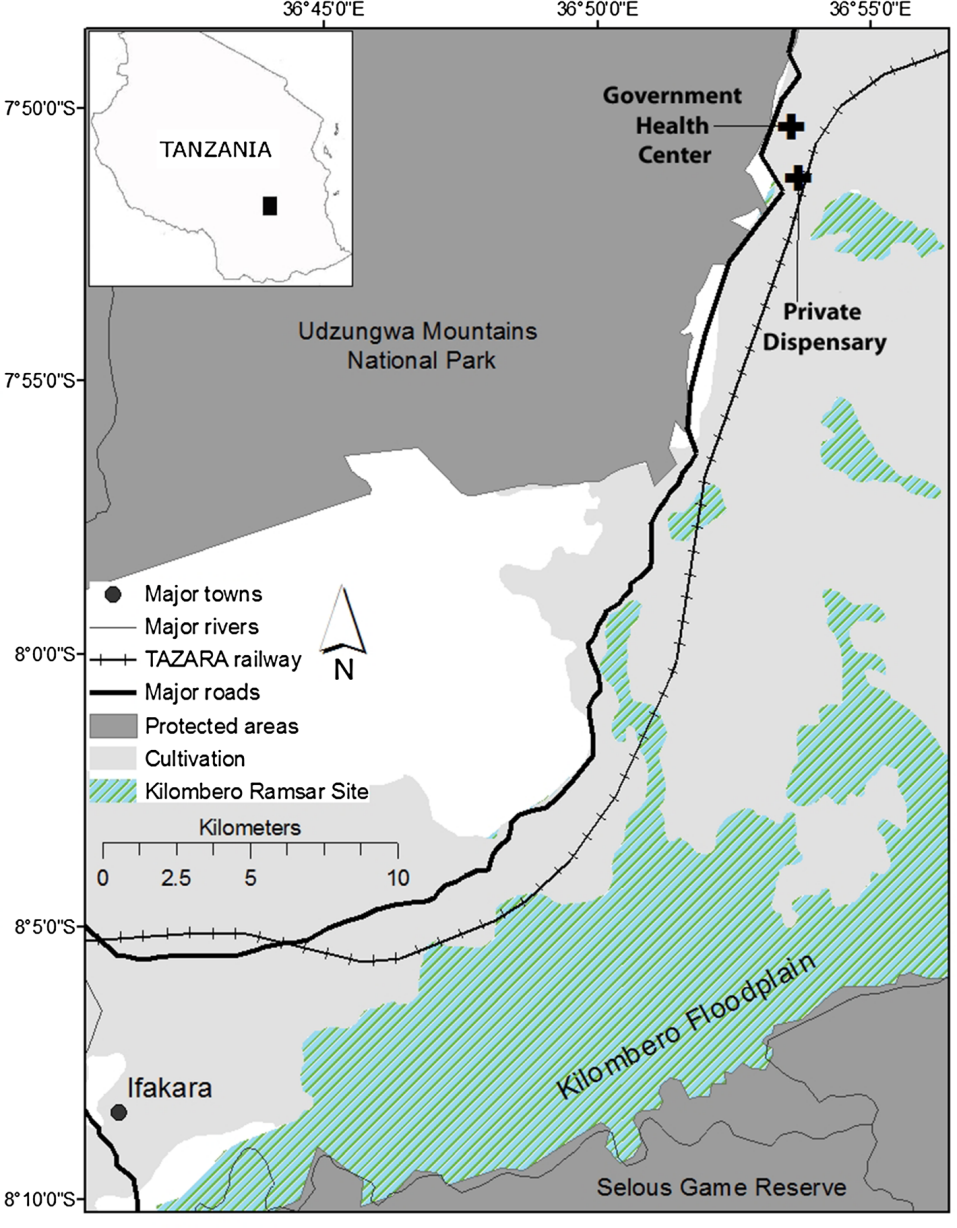
Map of study region indicating location of the two health facilities.

Malaria case data were collected for patients at two health facilities in Kilombero District, Morogoro, southern Tanzania. These facilities are a private dispensary in the village of Mwaya (S 07°51’17.4”, E 036°53’39.9”) and a government-owned public health centre in the village of Mang’ula (S 07°50’19.9”, E 036°53’32.8”). The two villages are 1.8 km apart, and adjacent to the Udzungwa Mountains National Park (Figure 1). *Plasmodium falciparum* is the only malaria species reported in this region [1].

According to the 2002 Tanzania Housing and Population Census, Mwaya has a population of 9,841 people and Mang’ula a population of 12,083 people [16]. The Kilombero Valley’s population is currently estimated at 321,611 people.

According to the National Park’s weather station, mean annual rainfall in this area ranges from 1,200-1,800 mm and follows a unimodal pattern falling from December to April; however, variability in this pattern has been observed in recent years (pers obs 2008-present).

### Study design

A direct comparison between RDTs and microscopy was made at the private dispensary from June to mid-August 2011. The results were used to estimate retroactively the levels of over-diagnosis for much of the previous three and a half years at the nearby public health centre, which had more extensive data on monthly malaria rates, saw more patients and had a wider demographic of patients.

### Study participants

At the private dispensary, eligible patients included any patient for whom the clinician judged a malaria diagnostic test necessary, based on case history and presenting symptoms. Between June and August 2011, 400 patients participated in this study and were tested with both microscopy and RDT.

The study population at the public health centre was made up of all patients who were tested for malaria during the time period for which malaria records were available, i e, from December 2007 until August 2011, excluding April 2008, September 2008 to March 2009, February 2010, and August 2010 to October 2010. Because of limited availability of malaria records at the public health centre, data during these months were never collected. All 9,175 patients tested with microscopy during this period were included in the retrospective study.

### Study protocol

At the private dispensary, results of RDTs (ICT Malaria Combo Cassette, ICT Diagnostics, Cape Town, South Africa) and blood slide microscopy were directly compared. ICT Malaria Combo Cassette Test is a rapid, *in vitro* diagnostic test for the detection of circulating *P*. *falciparum* antigens (HRPII antigen); an antigen that is common to all five species of human malaria is aldolase antigen [17]. According to WHO RDT Product Testing (Round 1) results from 2008, the detection rate of the ICT Malaria Combo Cassette Test for identifying *P*. *falciparum* is 86% at low parasite density (<200 parasites/*μ*l) and 100% at a higher parasitaemia (2,000-5,000 parasites/*μ*l). This test is reported to have a *Plasmodium vivax* detection rate of 0% at low parasite density (<200 parasites/*μ*l) and 95% at a higher parasitaemia (2,000-5,000 parasites/*μ*l) [17]. Given the high sensitivity and specificity of RDTs, and because of the known inaccuracy of routine microscopy in many rural clinics, the RDT was considered the gold standard for the purposes of this study.

Each patient tested at the private dispensary completed a short questionnaire providing sociodemographic characteristics, medical history, information on malaria prevention measures, and information on the frequency and types of anti-malarial treatments used (see Additional file 1). After testing with both microscopy and RDTs, patients returned to the clinician with both laboratory results and a final clinical diagnosis was made by the attending doctor and recorded by the study team.

At the public health centre, RDTs had been introduced by another research project as the sole diagnostic method for malaria in March 2011, in accordance with national policy of scaling up of RDTs at the country level. Prior to this introduction, microscopy was the sole diagnostic method. Because this clinic had ceased to use microscopy at the time of this study, the clinic did not at any point use RDTs and microscopy simultaneously. Therefore, given potential seasonal fluctuations in malaria burden, it was not possible to compare RDT results to microscopy results. Instead, a side-by-side comparison was done between the data collected at the private dispensary and the data collected previously at the public health centre. Hard copies of patient malaria records collected before and after RDT introduction were digitized to estimate the potential overuse of anti-malarials following diagnosis using microscopy. These records included patient age, sex, address, and the blood slide results (for patients tested prior to March 2011) or RDT results (for patients testing from March 2011 onwards).

### Analysis of cost-effectiveness

At the public health centre, regardless of the type of diagnostic test used, the cost to the patient for malaria testing was 0.25 USD (500 Tanzanian Shillings, at 2011 conversion rates). The cost to the government of using RDT kits was 1.44 USD, adjusted for the price of the ICT Combo Cassette test (0.52 USD/test) (adapted from [18]); the cost of using microscopy was estimated at 0.59 USD [18]. These costs to the government include labour costs associated with each test and were used to calculate the average monthly costs spent by the government on malaria diagnostics. The average number of each diagnostic tests preformed was also calculated. Based on observations at the clinic, it was assumed that all patients diagnosed with malaria using either an RDT or microscopy received and used anti-malarial drugs. In conjunction with the data collected from the direct comparison at the private dispensary, it was possible to calculate: 1) an estimate of government expenditure on unnecessary anti-malarial treatments; 2) the cost to the government of providing subsidized RDTs; and, 3) the net savings resulting from accurate malaria diagnosis.

### Statistical analyses

All analyses were undertaken using R version 2.13.1 [19]. Only patients with complete data including age, sex, and malaria test results were included in the analysis. The breakdown of age and sex was tabulated for the data from each of the two health facilities. A binomial exact test was used to determine the rates of positive and negative microscopy results, and the rates of positive and negative RDTs at both facilities. Using the epi.tools package in R, the specificity, sensitivity, negative predictive values, positive predictive values, 95% confidence intervals, and the apparent and estimated over-treatment with anti-malarials was calculated for the private clinic. RDTs were considered the gold standard for this analysis. Using the retrospective design, the extent of over-treatment with anti-malarials and the cost-effectiveness of the introduction of RDTs were calculated for the public health centre. Because a large proportion of patients using clinics were children, a non-parametric regression was used in which subsequent age categories were double the size of the previous age category in order to allow a better representation of prevalence of malaria at a young age (one month, three months, six months, one year, two years, four years, eight years, 16 years, 32 years, 64 years, and 64+ years). Association between malaria prevalence and variables such as age and rainfall were tested with Pearson’s product–moment correlation.

### Ethical considerations

Prior to participating in this study, each patient provided informed written consent. Ethical approval for this study was obtained from the Tanzania National Institute for Medical Research (NIMR/HQ/R.8a/Vol. IX/1165) and the Princeton University Institutional Review Board (IRB # 5374).

## Results

### Study population

At the private dispensary, 195 children and 205 adults (400 total; 47% males and 53% females, mean age = 32.6 years, median age = 32 years, SD = 23.04 years) were tested for malaria using both microscopy and RDTs. Of these, 317 patients (79%) tested positive with microscopy and 57 (14%) tested positive with RDTs (Table 1).

**Table 1:** Breakdown of the sample population in the public and private facilities including sample size, age, microscopy and RDT results

	**Mang’ula (Public)**	**Mwaya (Private)**
**Sample Size (N)**	11049	400
**Age**		
Median age	10 (SD = 16.54)	32 (SD = 23.04)
Children	6797	195
Adult	4152	205
**Sex**		
Male	3963 (36%)	188 (47%)
Female	6986 (64%)	212 (53%)
**Microscopy**	n = 9175	n = 400
Positive	6769	317
Negative	2406	83
Prevalence	74%	79%
**RDT**	n = 1774	n = 400
Positive	88	57
Negative	1686	343
Prevalence	5%	14%

At the public clinic, data were acquired from 10,949 malaria cases (6,797 children and 4,152 adults, 36% males and 64% females). The mean age of patients testing for malaria at the public clinic was 15.5 years, median age was 10 years, and the standard deviation was 16.54 years. These patients were tested for malaria using either a microscopy test or an RDT (post March 2011). Out of 9,175 patients, 6,769 (74%) tested positive with microscopy and 88 out of 1,774 patients (5%) patients tested positive with RDTs (Table 1).

**Figure 2:**
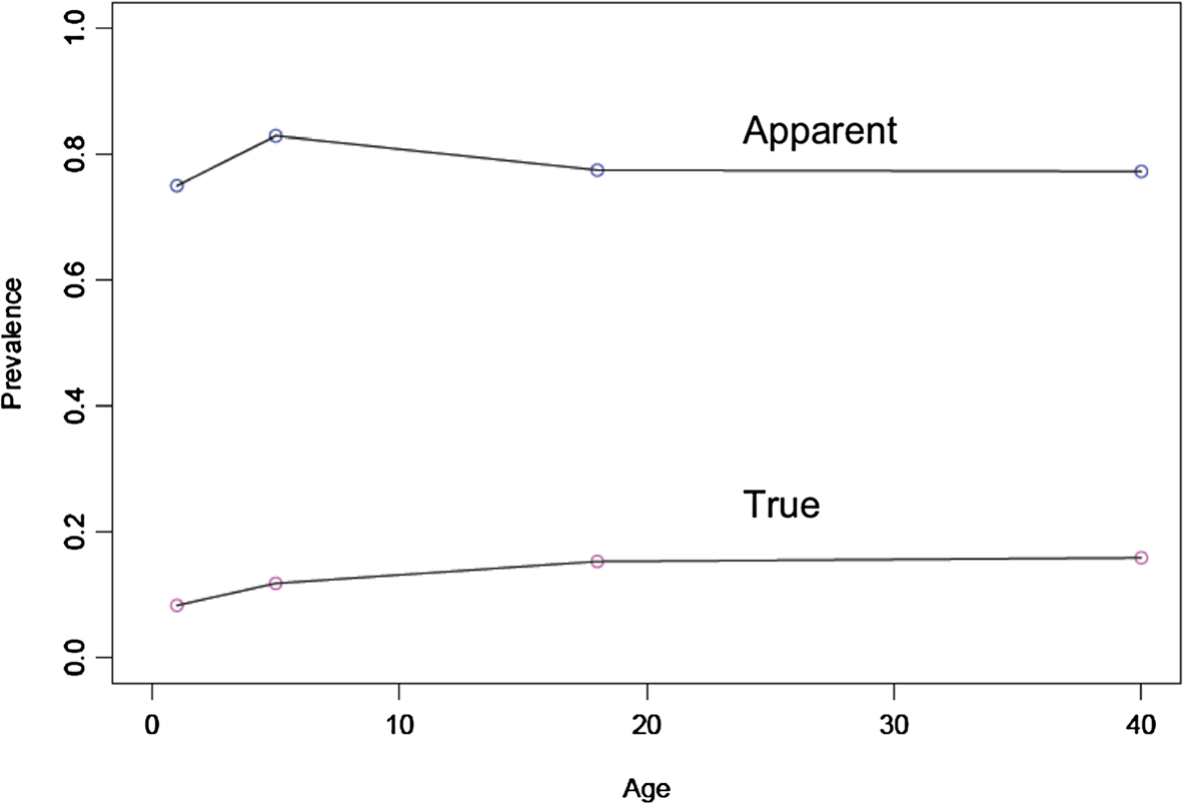
Apparent and true prevalence of malaria at the private clinic in Mwaya.

Table 2 shows the sensitivity, specificity, positive predictive values (PPV), and negative predictive values (NPV) of microscopy at the private dispensary using RDTs as the gold standard. Using the PPV and NPVs, the percentage of patients visiting the private clinic with malaria could be calculated demonstrating an apparent malaria prevalence of 78% and true malaria prevalence of 14% (Figure 2). This indicates that when using microscopy as the sole diagnostic test, malaria is being over-diagnosed by 64%. All (100%) patients at the private clinic reported regular usage of bed nets.

**Table 2:** Sensitivity, specificity, positive predictive values (PPV), and negative predictive values (NPV) of microscopy by age category, as well as the overall sensitivity, specificity, PPVs and NPVs using RDTs as the gold standard

**Age**	**Sensitivity**	**Specificity**	**PPV**	**NPV**
	**(95% CI)**	**(95% CI)**	**(95% CI)**	**(95% CI)**
Less than 1	1.00	0.27	0.11	1.00
	(0.01 – 1.00)	(0.06 - 0.61)	(0.00-0.48)	(0.19 – 1.00)
1-5	1.00	0.19	0.14	1.00
	(0.55 – 1.00)	(0.11 -0.31)	(0.07 - 0.25)	(0.66 – 1.00)
6-18	0.88	0.24	0.17	0.92
	(0.64 - 0.99)	(0.16- 0.34)	(0.10-0.27)	(0.74 -0.99)
19+	1.00	0.27	0.20	1.00
	(0.82 – 1.00)	(0.20-0.35)	(0.14 -0.28)	(0.87 – 1.00
Overall	0.96	0.25	0.18	0.98
	(0.87-1.00)	(0.20 - 0.30)	(0.14- 0.22)	(0.92 – 1.00)

### Malaria prevalence and age

**Figure 3:**
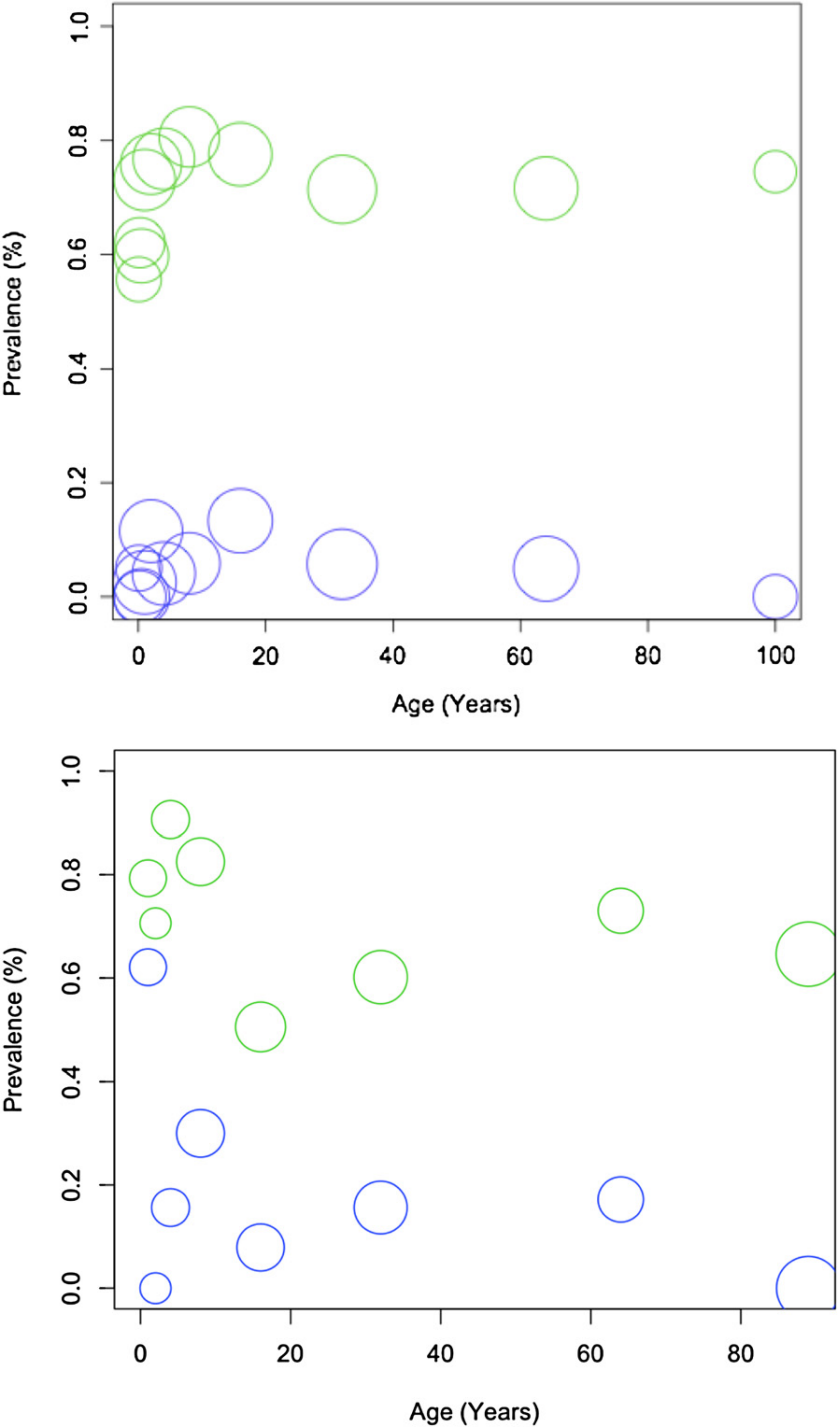
**Scatter plot of RDT (blue) and microscopy (green) prevalence in the public health centre (top) and private dispensary (bottom).** In the public health centre, either RDTs or microscopy was used to diagnose malaria. In the private dispensary, both RDTs and microscopy were performed on the same patient. The sizes of the circles represent the total number of patients, in that particular age category, who were tested.

Apparent malaria prevalence was higher when using microscopy than when using RDTs in both the private dispensary and public health centre and for each age category (Figure 3). There was no association between apparent or true prevalence and age of patient at either the private dispensary or the public health centre (Private: t = −0.92, df = 9, p = 0.38, r = −0.29; Public: t = 0.61, df = 9, p = 0.55, r = −0.20).

### Anti-malarial treatment at the private clinic

Of the anti-malarial medications used by patients in the private dispensary, artemether-lumefantrine (ALu) was the most frequently used medication (81%). At the time of this study, ALu cost 0.31 USD for patients zero to three years old, 0.63 USD for patients four to eight years old, 0.94 USD for patients nine to 14 years old, and 1.26 USD for patients 15 years and older. Metakelfin, or sulphadoxine-pyrimethamine combination therapy, was the second most frequently used medication (13%; 1.89 USD). Finally, quinine was the least frequently used medication (5%, 3.78 USD) (Table 3). As a result of AFMm, the cost per treatment for ALu to first-line buyers is 0.33 USD with a co-payment by the Global Fund of 0.32 USD per treatment [20].

**Table 3:** Prices and frequency of anti-malarial treatments purchased at the pharmacy connected to the private clinic

**Medication**	**Frequency (%)**	**Price (USD)**
ALu	81%	Age: 0-3: 0.31
		4-8: 0.63
		9-14: 0.94
		15+: 1.26
Metakelfin	13%	1.89
Quinine	5%	3.78

### Analysis of cost-effectiveness

At the public health centre, the average monthly costs (including labour) of performing diagnostic tests with microscopy were estimated at an average of 208 USD spent by the government on 352 microscopy tests per month from December 2007 to February 2011 (excluding April 2008, September 2008 to March 2009, February 2010, and August 2010 to October 2010). Conversely, an average of 425 USD was spent by the government clinic on 295 RDTs per month during the six-month period of March 2011 to August 2011.

For the purpose of the cost analysis, the mean age of patients (15.5 years) testing for malaria at the public health centre was used to determine the average price spent on ALu by patients. This amounted to an average of 328 USD spent on ALu per month since December 2007 when microscopy was used to diagnose malaria, and an average of 15 USD/month spent on ALu since the introduction of RDTs in March 2011. Because the true prevalence of malaria appears to be only 14% in this area, 5,485 of the 6,769 patients testing positive with microscopy received malaria medication for febrile illness caused by reasons other than malaria, resulting in the over-prescription of anti-malarials, improper treatment of these patients, and an extra 265 USD/month spent on anti-malarials. Thus, even with the added costs of RDTs, the introduction of RDTs resulted in a net savings of 96 USD/month. Using microscopy, there were two cases of false negatives, but overall, the number of false negatives was negligible compared to the number of false positives.

## Discussion

There was a marked difference between the number of positive malaria cases diagnosed with microscopy tests and with RDTs. This difference suggests that malaria is being over-diagnosed by 64% with microscopy in this rural region of Tanzania. This study was not random-controlled; therefore, some sample bias was inherent given that only patients living in the vicinity of these two clinics, and sick enough to seek medical treatment, were included in the study. Additionally, cautions are needed when extrapolating results of retrospective data as potential differences in the ecology and epidemiology of malaria may exist between the two neighbouring villages, although given their close proximity, this is highly unlikely. For example, no association was found between malaria prevalence and rainfall, at either clinic [Pearson’s test]. Additionally, while the prescription practices of health care workers after a positive and negative RDT result could certainly affect the assumed cost-effectiveness of the introduction of RDTs, this study did not collect these data. Future studies should investigate the effects of these practices on the deployment of RDTs.

The introduction of RDTs at the public clinic proved to be cost-effective, resulting in a net saving of 96 USD/month for the Tanzanian government. As the estimated RDT-diagnosed prevalence of malaria in this study was 14%, RDT introduction is recommended given WHO findings that RDTs are predicted to be cost-effective in prevalence areas of less than 20% [21].

The findings of malaria over-diagnosis in this study are consistent with past studies that have found that, because clinicians often perceive routine microscopy as unreliable, with the exception of tests done on children in high malaria transmission areas, most adults and children treated for malaria at almost every malaria transmission level show no evidence of parasites on carefully examined research slides [22,23]. Of course, the accuracy of the RDT should also be considered – while used as the gold standard in this study, no RDT is 100% sensitive and specific. In this particular study, there were two instances where the RDT result was negative but the microscopy was positive. Further testing of this particular RDT in the Kilombero Valley would help elucidate the exact efficacy of this RDT in this region.

### Policy recommendations

In April 2012, WHO launched the “T3: Test. Treat. Track.” Initiative, which urges countries to scale up diagnostic testing, provide quality-assured treatment, and follow-up surveillance for malaria [24]. Both for malaria control reasons and because ACT is expensive, it is important to target the population truly infected with malaria. RDTs are a cost-effective, highly sensitive and specific test in recognizing *P*. *falciparum*. Some RDTs, including the ICT Combo Cassette Test used in this study, have the capacity to detect other *Plasmodium* species, but these are more expensive. Therefore, understanding which species of malaria exist in specific areas can enable the selection of the proper RDT to maximize cost-effectiveness. In the case of the Kilombero Valley, where only *P*. *falciparum* has been detected, the introduction of a *P*. *falciparum*-only RDT would result in even greater savings. Implementing a policy aimed at decreasing the current over-diagnosis of malaria in the Kilombero Valley and follow-up monitoring cases would certainly improve the quality of life of people living in this malaria-endemic region.

According to the World Malaria Report of 2011, with current prices of RDTs and ACT, and perfect compliance with test results, savings resulting from not over-prescribing ACT could amount to $68 million (USD) in the public sector in the WHO African Region [1]. If a policy introducing RDTs to all clinics (public and private) was implemented to increase the reliability of diagnostic testing of febrile patients, and quality assurance measures were in place to ensure that both the public and the private sectors would abide by the policy, savings would likely be even greater. This is consistent with previous studies that have found public and private sectors are important complementary sources of malaria treatment in rural Tanzania [25]. If the government was able to provide universal RDTs to both the private and public sector, and if the government was able to better regulate the distribution, prescription, and sale of anti-malarial drugs, savings and improved care for all febrile patients in malaria-endemic areas would follow.

In November 2012, the Global Fund Board approved the integration of the AMFm into core global fund grants to continue subsidizing ACT [26]. While it is very important that quality ACT is available for proper treatment of febrile cases attributed to malaria, if reliable diagnostic methods are not in place, it is difficult to ensure that only those patients who actually have malaria receive ACT. Therefore, keeping in mind the WHO “T3. Test. Treat. Track.” initiative, if some funds from the AMFm subsidy on ACT were shifted to subsidizing RDTs, patients would receive accurate diagnoses and as a result, less ACT would be needed.

## Conclusions

This study showed that malaria is being over-diagnosed by 64% with microscopy in this rural region of the United Republic of Tanzania. Due to ongoing concerns regarding drug resistance to anti-malarial treatments, it is important that only patients who test positive for malaria use anti-malarial drugs. RDTs are accurate parasitological-based tests that provide a reliable option for malaria diagnosis in resource-limited areas. If clinicians properly use and act upon the results of RDTs, the introduction of RDTs to health facilities in Tanzania would be extremely cost-efficient. A policy dictating that private and public clinics work together and stay dedicated to properly diagnosing patients using RDTs, in conjunction with AMFm continuing to subsidize the costs of anti-malarials, will decrease the likelihood of drug resistance and result in proper treatment of non-malarial febrile illnesses.

## Competing interests

The authors declare that they have no competing interests.

## Authors’ contributions

All authors participated in the conception and design of the study. KH carried out the data collection, KH, KN, CS, and AD carried out the data and statistical analysis. All authors read and approved the final manuscript.

## Supplementary Material

Additional file 1 Questionnaire used at the private clinic. Click here for file
