# Supplementary material for: Over-diagnosis of malaria by microscopy in the Kilombero Valley, Southern Tanzania: an evaluation of the utility and cost-effectiveness of rapid diagnostic tests
Source: Malar J. 2013 May 10;12:159. doi: 10.1186/1475-2875-12-159 (PMC3689637; doi:10.1186/1475-2875-12-159)
Supplement: Additional file 1 — Questionnaire used at the private clinic. [file 1475-2875-12-159-S1.pdf]

Supplemental Material: Questionnaire used at the private clinic

ID No. ________________ Date: ____/____/________ dd/mm/yyyy
Time: ________________

PATIENT DETAILS

AGE ____________ (in yrs) SEX M ❑ F ❑

CURRENT SYMPTOMS

Duration of current illness Symptoms (current)
_____days

Temperature _________ ^{0}C

	No	Yes
Fever	❑	❑
Chills	❑	❑
Sweating	❑	❑
Headache	❑	❑
Other	❑	❑

Specify:________________________

RECENT CLINICAL HISTORY

Symptoms (past month)

	No	Yes
Fever	❑	❑
Chills	❑	❑
Sweating	❑	❑
Headache	❑	❑
Other	❑	❑

Specify:________________________

TREATMENT

(In the past month, what type of medicines have you taken?)

	No	Yes
Chloroquine	❑	❑
Sulfadoxine/Pyrimethamine	❑	❑
Primaquine	❑	❑
Alu	❑	❑
Other	❑	❑

Specify:________________________

QUESTIONS

Did you immigrate to Mwaya? If so, from where did you immigrate?
____

Do you have a bed net at home?
____

If you have a bed bet, do you regularly sleep under the bed net at nighttime?
____

If you have a bed net, how often do you wash it? ____________________________

Mang'ula RDT?________________________
